# NSAIDs and Acute Pancreatitis: A Systematic Review

**DOI:** 10.3390/ph3030558

**Published:** 2010-03-10

**Authors:** Raffaele Pezzilli, Antonio Maria Morselli-Labate, Roberto Corinaldesi

**Affiliations:** Department of Digestive Diseases and Internal Medicine, Sant’Orsola-Malpighi Hospital, Via Massarenti 9, 40138 Bologna, Italy; Email: antonio.morselli@unibo.it (A.M.M.-L.); roberto.corinaldesi@unibo.it (R.C.)

**Keywords:** acute pancreatitis, cytokines, inflammation, arachidonic acid, prostaglandins, leukotrienes, phospholipase A2

## Abstract

The resulting pain is the main symptom of acute pancreatitis and it should be alleviated as soon as possible. NSAIDs are the first line therapy for pain and they are generally administered to acute pancreatitis patients upon admission to the hospital. In addition, these drugs have also been used to prevent post-endoscopic cholangiopancreatography (ERCP) acute pancreatitis. On the other hand, there are several reports indicating that NSAIDs may be the actual cause of acute pancreatitis. We carried out a literature search on PubMed/MEDLINE; all full text papers published in from January 1966 to November 2009 on the use of NSAIDs in acute pancreatitis were collected; the literature search was also supplemented by a review of the bibliographies of the papers evaluated. Thus, in this article, we will systematically review the current literature in order to better illustrate the role of NSAIDs in acute pancreatitis, in particular: i) NSAIDs as a cause of acute pancreatitis; ii) their use to prevent post-retrograde ERCP pancreatitis and iii) their efficacy for pain relief in the acute illness of the pancreas.

## 1. Introduction

Pain is the main feature of acute pancreatitis (AP) and the majority of patients are admitted to the hospital for this reason. Management of AP is limited to supportive care and the treatment of complications when they develop. These patients require regular hospital admission, fluid administration, bowel rest and pain management. 

There are no extensive studies on the pharmacological control of pain in acute pancreatitis patients [1,2,3,4], which is quite surprising given the importance of this symptom during the course of the disease. There is also a lack of evidence regarding the degree of efficacy of the various pharmacological substances used to treat the different forms of acute pancreatitis. On the other hand, there are several reports concerning the possibility that non-steroidal anti-inflammatory drugs (NSAIDs) may actually induce acute pancreatitis [5,6,7,8,9,10,11,12,13,14,15,16,17]. NSAIDs have also been used to prevent retrograde endoscopic cholangiopancreatography (ERCP)-induced acute pancreatitis [18]. Thus, in this article, we will systematically review the current literature in order to better illustrate the role of NSAIDs in acute pancreatitis, in particular i) NSAIDs as a cause of acute pancreatitis; ii) their use in preventing post-ERCP pancreatitis and iii) their efficacy for pain relief in the acute illness of the pancreas.

## 2. Literature Search

A search was made using the U.S. National Library of Medicine National Institutes of Health PubMed/MEDLINE database in order to select the data existing in the literature on NSAIDs and acute pancreatitis covering the period from January 1966 to November 2009. The Medical Subject Headings (MESH) terms used were “Anti-inflammatory Agents, Non-Steroidal” (explanatory variable) and “Pancreatitis” or “Pancreatitis, Acute Necrotizing” (outcome variables). We identified additional studies through a physical search of bibliographies from primary studies, review articles and key journals, and through contacts with experts in the field. For studies with multiple re-analyses, only the most recent article with the largest population was chosen. The investigators independently screened all articles for those that met broad inclusion criteria. A total of 92 citations were found [1,5,6,7,8,9,10,11,12,13,14,15,16,17,18,19,20,21,22,23,24,25,26,27,28,29,30,31,32,33,34,35,36,37,38,39,40,41,42,43,44,45,46,47,48,49,50,51,52,53,54,55,56,57,58,59,60,61,62,63,64,65,66,67,68,69,70,71,72,73,74,75,76,77,78,79,80,81,82,83,84,85,86,87,88,89,90,91,92,93,94,95]. Of these 92 papers, 79 were excluded [5,6,7,8,9,10,11,12,13,14,15,16,17,19,20,21,22,23,24,25,26,27,28,29,30,31,32,33,34,35,36,37,38,39,40,41,42,43,44,45,46,47,48,49,50,51,52,53,54,55,56,57,58,59,60,61,62,63,64,65,66,67,68,69,70,71,72,73,74,75,76,77,78,79,80,81,82,83,84] because they contained data regarding diseases other than acute pancreatitis and used drugs other than NSAIDs, or were case reports or letters, comments and review articles not containing data which met the aims of the study or were duplicate publications. Thus, 13 papers were considered for the present study; they were four studies [92,93,94,95] regarding the incidence of acute pancreatitis in NSAID consumers, five randomized studies [18,87,88,89,90] regarding the prophylactic use of NSAIDs in preventing post-ERCP pancreatitis together two meta-analytic studies on this topic [85,86] and two studies regarding the efficacy of NSAIDs in the pain control in acute pancreatitis patients [1,91].

## 3. NSAIDs as Inducers of Acute Pancreatitis

There are numerous case reports on the association of the use of indomethacin, piroxicam, ketoprofen, naproxen, rofecoxib and celecoxib with acute pancreatitis. Even if these reports are of importance in pointing out the possible risks of this class of drugs, we should stress the fact that NSAIDs are widely used in the general population of developed countries. A study was carried out in Italy between March and September 2002 evaluating the use of generic drugs in a free-living population demonstrated that 20% of the NSAID users were over 65 years of age and 18% were chronic users (daily or frequent use for more than six months). NSAID use was significantly higher in women, both for overall and for chronic use. The older age groups showed an increasing risk of chronic NSAID use because the presence of headache (25%), osteoarticular pain (19%), unspecified pain (15%) and osteoarthrosis (9%). More than 50% of all the NSAIDs were prescribed by physicians whereas about 44% were taken as self-treatment or following the advice of a pharmacist, relative, friend, *etc.* Thus, two studies exploring the frequency of acute pancreatitis during the course of NSAID assumption found that this phenomenon is negligible: Ibanez *et al*. [95] studied a total of 48,678 hospitalized patients using the medical records and 554 (1.1%) were diagnosed as experiencing an adverse drug reaction. After excluding upper gastrointestinal bleeding (226 cases) and certain bone marrow blood dyscrasias (42 cases), 286 patients with drug-induced events leading to hospital admission were identified in two years. Six cases of drug-induced pancreatitis were found: three related to diclofenac, one to oral contraceptives, one related to clomifene, and the remaining one to chlortalidone. In addition, biliary lithiasis was identified in two of the patients with diclofenac-related pancreatitis and in the clomifene-related case, and although the stones were not located in the common bile duct, this finding could also explain the biliary etiology of pancreatitis. In conclusion, only one case were considered by the authors related to NSAIDs use. These data were confirmed by a population-based study carried out in 1993 [93]. In this latter study the authors found that a causal relationship of pancreatitis attributed to piroxicam was found in only one case out of 100,000 users of diclofenac, naproxen or piroxicam. On the contrary, in a case-control study carried out in Sweden in 2002 [94], among 2,453 acute pancreatitis patients and 2,245 controls, the authors found that only one case and three controls had taken indomethacin, but 25 cases and 36 controls had taken diclofenac and, in a multivariate analysis, the adjusted odds ratio (OR) was 2.1 (95% CI: 1.2–3.4) for the use of NSAIDs between cases and controls. Furthermore, in a population-based case-control study including 3,083 cases of acute pancreatitis and 30,830 population controls [92], it was found that 0.7% of the cases and 0.4% of controls were current users (within 90 days before admission into the study) of celecoxib and 0.6% of cases and 0.4% of controls were former users (within 91 to 365 days before admission into the study) of this drug; 0.6% of the cases and 0.4% of controls were current users of rofecoxib and 0.8% of cases and 0.4% of controls were former users of rofecoxib; finally, 18.2% of the cases and 7.4% of controls were current users of NSAIDs and 11.9% of cases and 9.8% of controls were former users of NSAIDs. Thus, the adjusted OR for other non-steroidal anti-inflammatory drugs was 2.7 (95% CI: 2.4–3.0) with a substantial variation in risk between the individual drugs; the highest risk was for diclofenac (OR 5.0, 95% CI: 4.2–5.9) and the lowest for naproxen (OR 1.1, 95% CI: 0.7–1.7). In conclusion, there is a risk for acute pancreatitis patients taking NSAIDs and, in clinical practice, it seems that naproxen should be the preferred analgesic for limiting the risk of development of acute pancreatitis.

## 4. The Prophylactic Use of NSAIDs for Preventing Post-ERCP Acute Pancreatitis

In various prospective studies, the frequency of post-ERCP pancreatitis ranges from 1 to 14%. After exposure to trigger events, injury to the gland occurs in an extremely rapid time interval. In experimental models of acute pancreatitis, it has been suggested that digestive enzyme activation might occur within the acinar cells and it has been shown that, in the early stages of acute pancreatitis induced by secretagogues or by diet, there is a co-localization of digestive enzymes and lysosomal hydrolases within large cytoplasm vacuoles; this co-localization mechanism might result in activation of the digestive enzyme. The trigger events which may determine the final effect of acute pancreatitis during ERCP is still unknown; mechanical, chemical, enzymatic, and microbiological factors may be involved as well as factors related to the patient and the physician [97]. Finally, the hypothesis of the activation of chemokines by endoscopic maneuvers as a cause of acute pancreatitis cannot be ruled out [97]. Recent studies [98,99,100,101] have indicated the usefulness of ERCP as a model for studying the early inflammatory response in acute pancreatitis. In their study, Kiviniemi *et al.* [99] found that, in uncomplicated cases, acute phase response determined by serum C-reactive protein levels was rare and did not parallel the serum amylase or lipase levels. However, Blanchard *et al.* [102] hypothesized that cytokines may be produced primarily by pancreatic parenchymal cells. Reasoning that the ductal epithelium is the cell type most likely to be exposed to noxious stimuli in common causes of pancreatitis, such as ERCP and passage of a gallstone, they examined the response of well-differentiated pancreatic ductal adenocarcinoma cell lines to stimuli known to stimulate cytokine production in other cells. CAPAN-1 and CAPAN-2 cells were incubated with endotoxins or TNF-alpha and the supernatant was assayed for production of IL-1, IL-6 and IL-8 by ELISA. The cells were assayed for activation of the transcription factor NF-kappa B by electrophoretic mobility shift assay. These authors found no detectable production of IL-1 by either cell line. CAPAN-1 cells had a concentration-dependent production of IL-6 and IL-8 in response to both endotoxins and TNF-alpha. CAPAN-2 cells had a concentration-dependent production of IL-6 and IL-8 in response to TNF-alpha. They had low level expression of IL-8 which was unaffected by any concentration of lipopolysaccharide (LPS) and no detectable production of IL-6 in response to LPS. On the basis of these findings, the authors concluded that pancreatic duct cells may play an active part in the pathogenesis of acute pancreatitis through the production of cytokines. More recently, we also found [103] that ERCP maneuvers significantly increase the serum levels of C-reactive protein, amyloid A and IL-6 in patients who did not develop acute pancreatitis, thus confirming the data of Blanchard *et al*. [102]. However, the administration of IL-10 in reducing the incidence of pancreatitis after therapeutic ERCP in humans is still under debate [104,105]. NSAIDs are potent inhibitors of prostaglandins, phospholipase A2 and neutrophil-endothelial interaction; all of these are believed to play an important role in the pathogenesis of acute pancreatitis [106,107,108]. NSAIDs are also inexpensive, easily administered and have a favorable risk profile when given as a one-time dose, making them an attractive option in the pharmacological prevention of post-procedural pancreatitis [86]. Five studies have been published in the last decade on the efficacy of NSAIDs in preventing pancreatitis induced by ERCP [18,87,88,89,90]. Three studies utilized diclofenac; in two studies, diclofenac was administered in the form of a suppository at a dosage of 100 mg immediately after ERCP [18,87] and, in the third study, diclofenac was given by mouth at a dosage of 50 mg 30–90 min before the ERCP and 4–6 h after the procedure. Another two studies utilized indomethacin [88,90], given as suppositories at a dosage of 100 mg before ERCP examination. All the studies except one [87] demonstrated that NSAIDs prevented post-procedural acute pancreatitis in a significant manner. Two subsequent meta-analyses [85,86] on this topic concluded that the widespread prophylactic administration of NSAIDs may significantly reduce the risk of acute pancreatitis after ERCP, resulting in major clinical and economic benefits. However, we should point out that even if the studies considered were randomized controlled trials, they had different modalities of ERCP procedures (e.g., number of cannulations, number of pancreatic duct injections, whether a sphincterotomy was performed) as well as in pharmacological manipulation (e.g., choice of drug, route of delivery, timing of administration); all these differences render the studies heterogeneous and suggest the need to plan more rigorous studies on this topic.

## 5. NSAIDs for Treating Pain in Acute Pancreatitis

The pathological activation of sensory neurons and inflammatory sequelae constitute what is known as neurogenic inflammation which appears to be important in many organ systems, including the pancreas. The destruction of the pancreatic parenchyma during acute pancreatitis quickly induces an inflammatory reaction at the site of injury. The initial cellular response involves the infiltration of polymorphonuclear leukocytes into the perivascular regions of the pancreas. Within a few hours, macrophages and lymphocytes accumulate and phagocyte-derived oxygen radicals participate in a primary injury to the pancreatic capillary endothelial cells. The increased microvascular permeability facilitates margination and extravascular migration of additional neutrophils and monocytes, amplifying the inflammatory process [109] and leads to many metabolic consequences including pain, fever, hypotension, acidosis and acute respiratory distress syndrome. Pain in acute pancreatitis is also due to the release of tachykinin substance P and calcitonin-gene-related peptide. Factors which stimulate primary sensory neurons include hydrogen ions, heat, leukotrienes, arachidonic acid metabolites, bradykinins and proteases, such as trypsin [110].

The mechanisms of action of NSAIDs come from their ability to inhibit cyclooxygenase-dependent prostanoid formation [111]. Other NSAID mechanisms which have recently been proposed, but have not been completely proven, include intracellular inhibition of phosphodiesterase, inhibition of bradykinin levels, and the uncoupling of G-protein-membrane protein interactions [112,113]. Other studies have suggested that the major analgesic mechanism for NSAIDs is through a central mechanism while the major anti-inflammatory mechanism occurs at a peripheral site of action [114].

**Figure 1:**
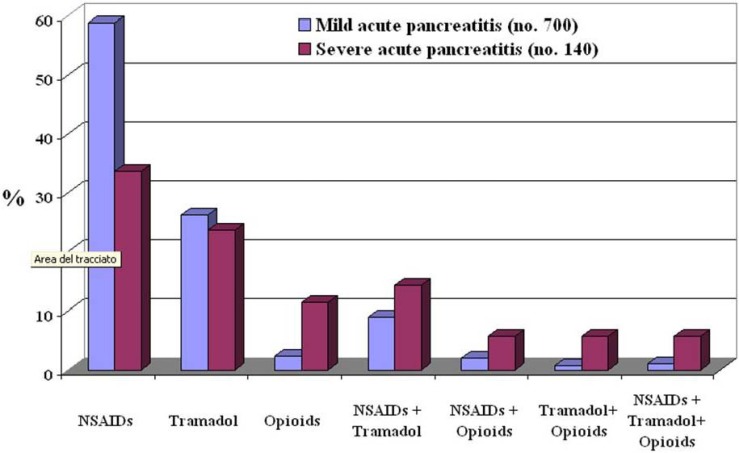
Type of analgesics administered to 700 patients with mild acute pancreatitis and to 140 with severe acute pancreatitis.

Only two studies exist in the literature on pain control with NSAIDs in patients with acute pancreatitis. The first paper was published in 1985 [1] and the other one in 2008 [91]. In the first controlled double-blind study, indomethacin, in the form of suppositories at a dosage of 50 mg twice daily for seven consecutive days, was compared to identical-looking placebo suppositories. There were 14 patients in the active treatment group and 16 in the placebo group. As expected, the number of days with pain and the number of opiate injections were significantly less in patients treated with indomethacin; most importantly, bleeding from the gastrointestinal tract was not seen in the indomethacin-treated group. In the second study, metamizole was compared to morphine; 8 patients with acute pancreatitis were randomized to receive 10 mg of morphine subcutaneously every 4 h and 8 patients received 2 g every 8 h of metamizole intravenously. Pain scores were recorded every 4 h during the first 48 h after admission using a visual analogical scale. Pethidine was also additionally administered as a rescue therapy. Seventy-five percent of the patients achieved pain relief in the metamizole group *versus* 37.5% in the morphine group within 24 h of hospitalization, but this difference was not statistically significant; the mean time for achieving pain relief was shorter in the metamizole group even if in a non-significant manner. At the end of the study (48 h after admission), 75% of patients achieved pain relief in the metamizole group *versus* 50% in the morphine group. Three patients in each group needed pethidine; two out of three achieved pain control in the metamizole group *vs.* none of the three in the morphine group. From the data of this study, intravenous metamizole shows a non-significant association with quicker pain relief than morphine given subcutaneously in acute pancreatitis but, most importantly, this drug seems to have the same efficacy as opiates in controlling acute pancreatitis pain. Currently, there is only one study which has evaluated how pain is routinely treated in patients with acute pancreatitis [115]. This study demonstrates that analgesics were graded according to the severity of the pain. In fact, patients with mild acute pancreatitis received mainly NSAIDs and tramadol whereas patients with severe pancreatitis received a high percentage of opioids or an association of analgesics comprising NSAIDs, tramadol and opioids. Data on analgesic administration were available in 840 of the 1,173 patients (71.6%) (Figure 1); NSAIDs were administered in 459 patients (54.6%), tramadol in 216 (25.7%), opioids in 32 (3.8%) and a combination of the various drugs in the remaining 133 (13.8%) patients. There was a significant difference in the type of analgesics administered between patients with mild and those with severe disease (P < 0.001); NSAIDs and tramadol were used more frequently in patients with mild disease whereas opioids associated with NSAIDs and or tramadol were used more frequently in patients with severe disease. The duration of analgesic treatment was significantly longer in patients with severe acute pancreatitis in comparison to those suffering from the mild form; NSAIDs: 3.0 ± 2.3 days in patients with mild acute pancreatitis and 6.7 ± 10.1 days in patients with severe acute pancreatitis (P < 0.001); tramadol: 3.2 ± 2.3 days in patients with mild acute pancreatitis and 8.0 ± 11.7 days in patients with severe acute pancreatitis (P < 0.001).

## 6. Conclusions

The answers to the questions posed as to whether NSAIDs may cause acute pancreatitis, whether their prophylactic use is able to prevent post-ERCP pancreatitis, and whether they are capable of controlling pain in acute pancreatitis are the following: 1) there is a risk for acute pancreatitis associated with the use of NSAIDs and, in clinical practice, it seems that naproxen should be the preferred analgesic in limiting the risk of development of acute pancreatitis; 2) both diclofenac and indomethacin may significantly reduce the risk of acute pancreatitis after ERCP resulting in major clinical and economic benefits and, finally, 3) NSAIDs are able to control the pain in acute pancreatitis patients. However, further clinical studies on the best NSAID to be used in clinical practice are needed. An example comes from the use of diclofenac; this is a drug largely used to treat pain in acute pancreatitis. It is useful in preventing post-ERCP pancreatitis, but it is considered the major NSAID responsible for inducing acute pancreatitis in the general population.
